# The moderating role of neighborhood disadvantage on the link between functional limitations and self-rated health

**DOI:** 10.1371/journal.pone.0283796

**Published:** 2023-04-05

**Authors:** Jeong Ha (Steph) Choi, Daniel H. Jung

**Affiliations:** 1 Department of Psychology, University of Wisconsin–Madison, Madison, WI, United States of America; 2 Department of Public Policy and Management, University of Georgia, Athens, GA, United States of America; Universidade Federal de Minas Gerais, BRAZIL

## Abstract

**Background:**

Self-rated health is an important health outcome and determinant of health. Improvements to our understanding on self-rated health could help design plans and strategies to improve self-rated health and achieve other preferred health outcomes. This study examined whether the link between functional limitations and self-rated health varies by neighborhood socioeconomic status.

**Methods:**

This study used the Midlife in the United States study linked with the Social Deprivation Index developed by the Robert Graham Center. Our sample consist of noninstitutionalized middle to older adults in the United States (n = 6,085). Based on stepwise multiple regression models, we computed adjusted odds ratios to examine the relationships between neighborhood socioeconomic status, functional limitations, and self-rated health.

**Results:**

Respondents in the socioeconomically disadvantaged neighborhoods were older, had higher percentage of females, non-White respondents, lower educational attainment, lower perceived neighborhood quality, and worse health status with greater number of functional limitations than those in socioeconomically advantaged neighborhoods. Results showed a significant interaction was found where neighborhood-level discrepancies in self-rated health was biggest among individuals with highest number of functional limitations (B = -0.28, 95% CI[0.53, -0.04], p = 0.025). Specifically, individuals with the highest number of functional limitations from the disadvantaged neighborhoods had higher self-rated health compared to those from advantaged neighborhoods.

**Conclusions:**

Our study findings highlight that neighborhood discrepancy in self-rated health is underestimated particularly among those with severe functional limitations. Moreover, when interpreting self-rated health status, values should not be taken face value, and should be considered together with the environmental conditions of where one resides.

## Introduction

Self-rated health reflects an individual’s overall perception of their own health [1], representing not only physical but also emotional and social aspects of health and well-being. As an important health outcome and determinant of health [2–8], improvements on our understanding on self-rated health could be beneficial to design plans and strategies to improve self-rated health, as well as achieving other preferred health outcomes. However, while studies have documented how individual- and neighborhood-level factors are independently associated with self-rated health [9–11], we have less information on the joint contribution of these factors on self-rated health. According to Jylhä [12], self-assessment of health is not only based on different types of information but also the environmental context in which the information is interpreted and processed. In this context, the same objective health status may not translate to the same subjective assessment of one’s health depending on one’s social and environmental surroundings. Studies have, in fact, found heterogeneity in self-reported health measures by socioeconomic status [13], and the relationship between biomarkers of health risk and self-rated health to vary by socioeconomic status [14]. Therefore, developing a further understanding of the interplay of individual and contextual factors on self-rated health is needed.

Functional limitations, defined as difficulties in carrying out daily activities people do to live independently and be integrated within the environment [15], are found to be a prominent condition in predicting self-rated health and health outcomes, with greater functional limitations predictive of worse self-rated health independent of chronic conditions [16]. People with functional limitations are in more need of health care services and help with activities of daily living than others. Moreover, the availability of community resources, caregivers support, and social support that they can access vary by neighborhood, further impacting individuals’ daily lives and health [17–19]. As such, how individuals rate their health status, especially for those with functional limitations, could depend on level of neighborhood disparity.

While the impact of neighborhood context on self-rated health for individuals with functional limitations is evident, how self-rated health among those with limited functional ability will differ by neighborhood status is unclear. Prior theories and findings provide two possible directions. According to the person-environment fit perspective [20], discrepancy between personal need and environmental resources may create additional problems beyond those from environment or personal preference alone. Based on this approach, one could expect the magnitude of the relationship between functional limitations and self-rated health to be worse among those from socioeconomically disadvantaged neighborhoods. Specifically, less disadvantaged neighborhoods are able to provide the personal needs for those with functional limitations, indicating a good level of person-environment fit. Conversely, disadvantaged neighborhoods are not able to fully support the needs of individuals with functional limitations. Such poor person-environment fit may become a source of additional stress for the individual, contributing to lower self-rated health. In line with this perspective, one study found that having a chronic condition was associated with poorer self-rated health among participants from more deprived areas compared to those living relatively more advantaged areas [21].

At the same time, differences in health expectations influenced by social factors such as socioeconomic status could underestimate neighborhood differences in the link between functional limitation and self-rated health. Social Comparison Theory suggests that self-evaluations are made based on comparing themselves to a reference group of their peers [22]. Thus, the health expectations of individuals from disadvantaged neighborhoods will be relatively low due to the lower average health of individuals in their community. Similarly, individuals from less disadvantaged neighborhoods will likely have a higher expectation for what is considered to be in good health. Furthermore, with greater limitations in daily functioning, the availability of resources and support may aggravate the gap in health expectations by neighborhood socioeconomic status, further contributing to neighborhood differences in subjective health. In line with this perspective, one study showed that the impact of health problems on self-rated health is found stronger among better educated individuals [23].

Following the two perspectives, the goal of this study was to examine the moderating role of neighborhood socioeconomic status (SES) on the link between functional limitations and self-rated health. The study utilized data from the Midlife in the United States (MIDUS) study linked with the Social Deprivation Index (SDI) developed by the Robert Graham Center [24]. Based on previous research, we tested the following hypotheses:

Hypothesis 1. Higher functional limitation is related to lower self-rated health.
Hypothesis 2a. The magnitude of the association between functional limitations and self-rated health will be *larger* for those in socioeconomically disadvantaged neighborhoods relative to advantaged neighborhoods (person-environment fit perspective).
Hypothesis 2b. The magnitude of the association between functional limitations and self-rated health will be *smaller* for those in socioeconomically disadvantaged neighborhoods relative to advantaged neighborhoods (social comparison theory).

## Material and methods

### Data and samples

We used data from the Midlife in the United States (MIDUS), a longitudinal survey of noninstitutionalized adults in the United States, focusing on MIDUS Wave 3 (MIDUS 3; 2013–2014) and MIDUS Refresher (MIDUS R; 2011–2014) Wave 1. The first wave of MIDUS (MIDUS1) was collected in 1995 and 1996 from a national random-digit-dial sample of adults between the ages of 25 to 74 (N = 7,108). Of the MIDUS 1 respondents, 4,963 respondents were re-interviewed approximately 9 years later (MIDUS 2), followed by a third wave of data collection with 3294 respondents in 2013–2014 (MIDUS 3). During the second wave of data collection, an African American sample (N = 592) from Milwaukee, Wisconsin was added to refine the MIDUS 2 sample. These individuals participated in a personal interview and completed a questionnaire paralleling MIDUS 2 assessments. 518 of these respondents were interviewed again between 2015–2016 as part of the third wave of data collection. The Refresher study was initiated from 2011 to 2014, which recruited a national probability sample of an additional 3,577 adults (age 25–74) to replenish and parallel the original MIDUS 1 baseline survey. The Refresher study also recruited a sample of 508 Milwaukee African American adults (age 25–64), to replenish the MIDUS 2 Milwaukee sample (2012–2013). Data collection for all MIDUS studies were approved by institutional review boards at the University of Wisconsin Madison, University of California Los Angeles, and Georgetown University, and all participants provided written informed consent. As we used data from an already existing data set, we were not involved in determining sample size nor involved in recruitment and data-collection stopping decisions. The datasets are publicly available in de-identified and anonymized forms and were downloaded from the MIDUS website for analysis.

Participants with no data on SDI, functional limitations, and self-rated health were excluded in the final analysis sample. The final sample consisted of 6085 respondents, of which 3208 were from the MIDUS 3 Project 1 sample, and 2877 were from MIDUS R. Provided that both MIDUS 3 and MIDUS R utilized the same data collection methodology with most approximate data collection periods, the datasets were combined to strengthen the power of the analyses.

### Measures

#### Self-rated health

Self-rated health was measured using a single-item scale (“Using a scale from 0 to 10 where 0 means "the worst possible health" and 10 means "the best possible health," how would you rate your health these days?”).

#### Functional limitations

Functional limitations were measured by using the measure of limitations in daily activities (α = .95). Respondents were given a list of activities of daily living including bathing or dressing yourself; climbing one flight of stairs; walking one block; lifting or carrying groceries; climbing several flights of stairs; bending, kneeling, or stooping; walking more than a mile; walking several blocks; vigorous activities (e.g., running, lifting heavy objects); and moderate activities (e.g., bowling, vacuuming). They were asked to report how much they experienced any difficulties performing each of the activities (1 = not at all; 4 = a lot). Functional limitation scores were computed in two ways: severity (range: 1–4), and count (5 groups: 0, 1–2, 3–5, 6–8, and 9–10). Severity reflected the degree to which one has difficulty performing daily activities on average, while count represented the number of different activities the individual has difficulty enacting in daily life. Thus, higher severity indicates the level of difficulty in performing daily activities, whereas higher count means the array of limitations in daily activities. Severity of functional limitations was computed for respondents who answered for at least seven items, and count was calculated for all respondents.

#### Neighborhood disadvantage

We used Social Deprivation Index (SDI) to estimate neighborhood socioeconomic status. Developed by the Robert Graham Center, SDI is a composite score of seven domains from the 2011–2015 American Community Survey: income, education, employment, housing, household characteristics, transportation, and demographics [24]. From a range of 0–100, higher SDI values indicated poorer neighborhood socioeconomic status. As MIDUS does not release any direct geographic identifiers in its publicly available datasets, SDI was linked to MIDUS through approval and process by the MIDUS administrative core. Quartiles of the SDI score were calculated based on the distribution of the score in our sample. We computed a binary indicator of neighborhood socioeconomic status using the third quartile value (SDI score = 71). Thus, SDI range of the higher SDI group was 71 to 100 (defined as socioeconomically disadvantaged neighborhoods), while that of the lower SDI group was 0 to 71 (defined as socioeconomically advantaged neighborhoods).

#### Control variables

Our analyses controlled for demographic variables: age (mean-centered), gender (male [Reference], female), race/ethnicity (White [Reference], Black, Other), educational attainment (high school/General Educational Development or lower [Reference], some college, Bachelor’s degree or higher), and number of chronic illnesses (mean-centered). Perceived quality of neighborhood (mean-centered) was also controlled for due to its link with health outcomes [25, 26]. Perceived neighborhood quality was assessed by rating four items on neighborhood safety and trust (e.g., “I feel safe being out alone in my neighborhood during the daytime.”) on a scale from 1(Not at all) to 4 (A lot). Scores were computed by averaging the scores of each item.

### Statistical plan

First, descriptive statistics were computed to examine sample characteristics both overall and by neighborhood disadvantage (disadvantaged neighborhoods vs advantaged neighborhoods). We used independent sample’s t-test and chi-square tests to assess differences in study variables by neighborhood disadvantage. Main analysis involved a stepwise multiple regression analysis. Specifically, we regressed self-rated health on neighborhood disadvantage (Model 1), neighborhood disadvantage and functional limitation count (i.e., 0; 1–2; 3–5; 6–8; 9–10; Model 2), their interaction (Model 3). All models included control variables (i.e., age, gender, race/ethnicity, perceived neighborhood quality, and number of chronic illnesses). A significance level of 0.05 was selected a priori. We also calculated adjusted self-rated health scores by functional limitations and neighborhood disadvantage. As a sensitivity analysis, we conducted the same full regression model (Model 3) with the interaction but replaced count with severity. We also conducted the same analyses with different cutoffs of SDI score to define neighborhood socioeconomic status (i.e., 25^th^ and 50^th^ percentile). Further, we conducted logistic regression using self-rated health as a binary measure (low [1–5] vs. high [6–10]) instead of a continuous measure. We performed all analyses using R version 4.04.

## Results

Table 1 presents demographic characteristics of the respondents in our analysis by neighborhood disadvantage. Respondents in the disadvantaged neighborhoods were older, had higher percentage of females (59.82%), high school or lower education (38.15%), some college education (34.69%), and non-White respondents (Black: 42.38; Other: 7.93%) than those in less disadvantaged neighborhoods. People in the disadvantaged neighborhoods also reported lower self-rated health (M = 6.98, SD = 1.82), lower perceived neighborhood quality (M = 3.11, SD = 0.67), higher functional limitation severity (M = 1.92, SD = 0.91), higher percentage of respondents with 6 or more functional limitations (6–8: 19.17%; 9–10: 25.27%), and higher numbers of chronic illnesses (M = 3.69, SD = 3.49) compared to others.

**Table 1:** Descriptive analysis of sample overall and by neighborhood disadvantage.

	Overall	Disadvantaged Neighborhoods	Advantaged Neighborhoods
	(N = 6085)	(SDI > 71; N = 1476)	(SDI < 71; N = 4609)
	M (SD)	M (SD)	M (SD)
Age^a^	57.92 (14.09)	55.76 (14.62)	58.62 (13.84)
Gender (%)^a^			
*Male*	44.77	40.18	46.24
*Female*	55.23	59.82	53.76
Education (%)^a^			
*HS/GED or lower*	27.73	38.15	24.39
*Some College*	30.38	34.69	29.00
*BA or higher*	41.89	27.16	46.61
Race/Ethnicity (%)^a^			
*White*	78.87	49.69	88.19
*Black*	13.48	42.38	4.26
*Other*	7.64	7.93	7.55
Perceived Neighborhood Quality ^a^	3.42 (0.56)	3.11 (0.67)	3.52 (0.47)
Self-rated Health ^a^	7.26 (1.67)	6.98 (1.82)	7.34 (1.60)
Functional Limitation Severity ^a^	1.76 (0.85)	1.92 (0.91)	1.7 (0.83)
Functional Limitation Count (%)^a^			
*0*	23.89	19.44	25.32
*1–2*	21.79	18.70	22.78
*3–5*	19.62	17.41	20.33
*6–8*	16.45	19.17	15.58
*9–10*	18.24	25.27	15.99
Number of Chronic Illnesses ^a^	3.16 (3.19)	3.69 (3.49)	2.98 (3.06)

^a^ Significant difference between Disadvantaged Neighborhoods and Advantaged Neighborhoods (p < 0.001).

Note: HS/GED = high school or General Educational Development; BA = Bachelor’s degree.

Table 2 reports estimates from stepwise multiple regression model analysis examining self-rated health on neighborhood disadvantage, functional limitation count, and their interaction. Model 1, based on neighborhood disadvantage but without functional limitations, shows neighborhood disadvantage was not significantly associated with self-rated health (B = -0.01, SE_B = 0.05, 95% CI[-0.12, 0.09], F(1, 5661) = -0.24, p = .81). When functional limitation count was added to the model (Model 2), functional limitation count was a significant factor related to self-rated health; compared to those with no functional limitation, those with 1 or more limitations indicated significantly lower self-rated health, and this difference increased with greater count (ts < -8.30, p < .001). When the neighborhood disadvantage and functional count interaction was entered in the model (Model 3), a significant interaction was found. Compared to those in the disadvantaged neighborhoods, those in advantaged neighborhoods demonstrated a bigger difference in self-rated health between none and 9–10 functional limitations (B = -0.32, SE_B = 0.13, 95% CI[-0.57, -0.07], t(5653) = -2.50, p = .013).

**Table 2:** Stepwise regression models regressing self-rated health by SDI group, functional limitation count, and their interaction.

	Model 1			Model 2			Model 3		
	(SDI Only)			(SDI & FL)			(SDI x FL Interaction)		
	B	SE_B	t	B	SE_B	t	B	SE_B	t
SDI group (Reference: Higher SDI group)	-0.01	0.05	-0.24	-0.07	0.05	-1.47	-0.02	0.10	-0.23
Functional limitation Count (Reference: No functional limitation)									
*1–2*				-0.45	0.05	-8.31***	-0.56	0.12	-4.70***
*3–5*				-0.86	0.06	-15.00***	-0.89	0.12	-7.40***
*6–8*				-1.32	0.06	-20.80***	-1.25	0.12	-10.45***
*9–10*				-2.15	0.07	-32.22***	-1.94	0.11	-16.90***
Functional limitation count x SDI group									
*1–2 x SDI group*							0.13	0.13	0.99
*3–5 x SDI group*							0.04	0.13	0.28
*6–8 x SDI group*							-0.10	0.13	-0.72
*9–10 x SDI group*							-0.32	0.13	-2.50*
Age (mean-centered)	0.08	0.02	3.84***	0.28	0.02	14.13***	0.29	0.02	14.32***
Gender (Reference: Male)	0.22	0.04	5.43***	0.32	0.04	8.78***	0.33	0.04	8.85***
Race/Ethnicity (Reference: White)									
*Black*	-0.06	0.07	-0.83	-0.01	0.06	-0.20	-0.03	0.06	-0.43
*Other*	-0.03	0.08	-0.38	0.01	0.07	0.13	0.01	0.07	0.15
Education (Reference: *HS/GED or lower*)									
*Some College*	0.09	0.05	1.64	-0.01	0.05	-0.14	0.00	0.05	-0.01
*BA or higher*	0.37	0.05	7.38***	0.10	0.05	2.23*	0.11	0.05	2.27*
Perceived Neighborhood Quality (mean-centered)	0.16	0.02	7.17***	0.08	0.02	3.99***	0.08	0.02	4.01***
Number of Chronic Illnesses (mean-centered)	-0.68	0.02	-32.80***	-0.41	0.02	-19.56***	-0.41	0.02	-19.68***

* < 0.05

** < 0.01

*** < 0.001.

Note: FL = Functional Limitations; HS/GED = high school or General Educational Development; BA = Bachelor’s degree.

**Fig 1:**
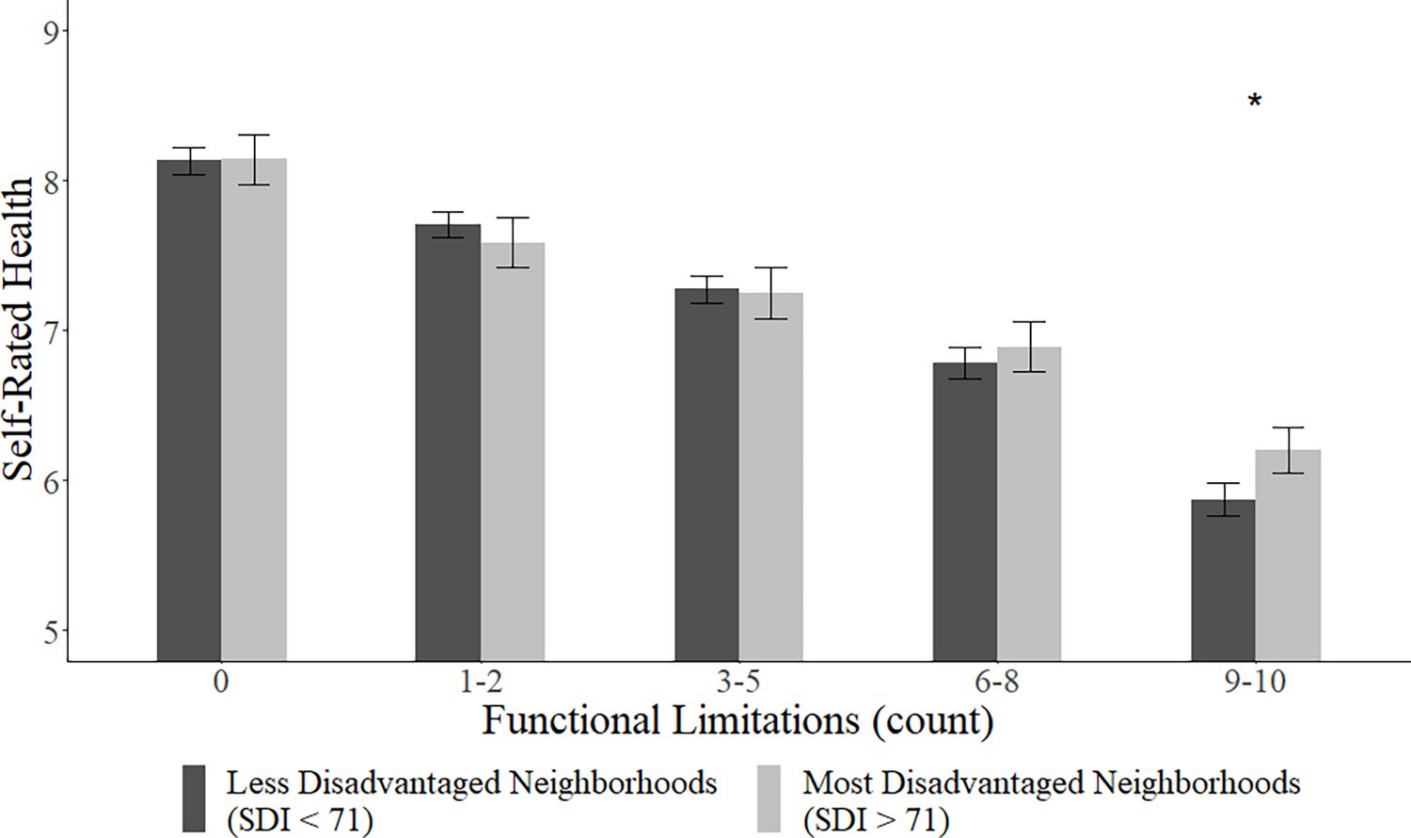
Association between functional limitations and self-rated health by neighborhood disadvantage. Note: Error Bars = 95% confidence interval; Dark grey bar = Disadvantaged neighborhoods (SDI > 71); Light grey bar = Advantaged neighborhoods (SDI < 71).

Fig 1 shows that, while there are no significant differences in self-rated health by neighborhood disadvantage when having no functional limitation, respondents in less disadvantaged neighborhoods reported lower self-rated health than their higher counterparts when having 9–10 limitations. Having one to two limitations was also related to lower self-rated health compared to none, and the gap increased as the number of functional limit increased. Demographic patterns were consistent across models; younger age, being female, higher level of education, higher perceived neighborhood quality, and higher number of chronic illnesses were related to lower self-rated health. There was no significant difference in self-rated health by race/ethnicity.

We further conducted sensitivity analyses using functional limitation severity, different SDI cutoffs (i.e., 25^th^ and 50^th^ percentile), or binary outcome (i.e., low [1–5] vs. high [6–10]). Results are presented as Supporting Information (S1–S3 Tables). When functional limitation severity was used in place of functional limitation count, we found a significant interaction of functional limitations by neighborhood disadvantage (S1 Table). Higher severity was related to lower self-rated health, and the magnitude of the association was bigger for respondents in advantaged neighborhoods (SDI < 71) than those in the disadvantaged neighborhoods (SDI > 71). To determine the region of significance where the group mean differences are and are not statistically different, we utilized the Johnson-Neyman technique [27]. Self-rated health differed by neighborhood disadvantage when severity was higher than 2.10, suggesting neighborhood gradients were mainly due to higher severity of functional limitations.

Furthermore, when tested with different SDI cutoffs and functional limitation count, results show that link between functional limitations and self-rated health do not vary by the changed cutoffs (S2 Table). Lastly, when self-rated health was further analyzed as a binary outcome (S3 Table), logistic regression results showed similar patterns of statistical significance to that of our main analysis, where we found a significant interaction of functional count (none vs. 9–10) and neighborhood disadvantage (OR = 0.46, 95%CI [0.23, 0.96], p = 0.032), similar to our main analyses. Such results imply that the link between functional limitations and self-rated health does not differ by how self-rated health was quantified within these analyses and current sample.

## Discussion

To the best of our knowledge, this is the first study to examine how the association between functional limitations and self-rated health varies by neighborhood socioeconomic status. Using data from a survey sample of noninstitutionalized adults in the United States, the study found the link between functional limitations and self-rated health was weaker among individuals of poorer neighborhood SES. That is, among those with most functional limitations, those living in poorer neighborhood SES tended to rate their current health as better compared to those of relatively better neighborhood SES. Such finding shows that neighborhood discrepancy in self-rated health is underestimated, and such pattern is particularly strong among those with a high number of functional limitations. These results provide evidence that similar degrees of functional limitations do not translate to the same level of self-rated health across socioeconomic status, and thus such subjective measures of health need to be interpreted with caution.

Our study finding is consistent with previous studies which have shown the association between functional limitation and self-rated health even after accounting for chronic conditions [16, 28, 29]. Self-rated health, as mentioned before, is a subjective indicator of health status integrating not only biological and functional status, but also environmental and social factors that affect the individual [1]. Also, functional limitations represent the restrictions and/or impairments in individual’s ability to perform activities for a normal life and further relating to reduced quality of life [30]. Taking these into account, the strong link between functional limitations with self-rated health further indicates how the latter is greatly reflective of one’s functional independence. Considering the importance of functional ability on self-rated health, efforts to expand interventions and programs targeting to support people with functional limitations could aim to improve self-rated health as a health outcome as well as an intermediate health indicator that affects other health outcomes such as mortality.

While we did find self-rated health to differ by neighborhood disadvantage, the significance disappears in the presence of control variables including educational attainment and perceived neighborhood quality. Specifically, the association disappeared with the addition of perceived neighborhood quality even without the presence of education in the model. This suggests that there is a large overlap between objective and subjective measures of neighborhood SES in their association with self-rated health, where the variance explained by perceived neighborhood quality on perceived health status overrides that of neighborhood SES. It is also in line with previous work which have examined the relationship of objective and subjective measures of neighborhood status [25]. Significant differences in perceived neighborhood quality by neighborhood disadvantage further supports this possibility (Table 1).

The significant interaction of functional limitations and neighborhood SES on self-rated health is in line with the idea that how individuals assess their health is conditional of context even with similar health conditions [12], and that health expectations are influenced by social factors beyond one’s current physical health [23]. Specifically for individuals with the highest number of functional limitations, we found higher self-rated health among individuals from the disadvantaged neighborhoods than others. This finding is in line with Jylhä’s conceptual framework on self-rated health, positing that environmental context affects how an array of information is interpreted and processed when individuals assess their health [12], and not in the direction expected based on prior work on health disparities by socioeconomic status.

Our findings also supported our hypothesis based on the Social Comparison Theory, suggesting that differences in health expectations underestimate the neighborhood discrepancy in self-rated health. The presence of relatively accessible resources and perceived social support [17–19] may influence individuals with severe functional limitations to compare their visibly poor physical status to their peers with much better health conditions. In contrast, those of relatively more disadvantaged neighborhoods are less likely to be exposed to the same support and resources, thus are less likely to consider their poor physical condition into how they rate their current health status. In fact, our sample respondents in the disadvantaged neighborhoods had greater physical issues including higher number of chronic illnesses and functional limitations, both in severity and number. With this in mind, the relatively higher subjective health status among those from lower neighborhood SES is likely less reflective of current functional impairment than respondents of relatively higher neighborhood SES. We also note that significant differences were largely driven by respondents with highest severity and count in functional limitations. This implies that, when considering the role of neighborhood context in how functional limitations and self-rated health are linked, we are likely to see greatest differences among those who with the most limitations in functional ability.

Sensitivity analyses also provide additional information on the role of neighborhood disparity on the link between functional limitations and self-rated health. Regardless of how functional limitations were measured, differences in self-rated health by neighborhood socioeconomic status appear to be underestimated among those with severe difficulties in daily functioning. Moreover, statistically non-significant interactions with lower SDI cutoffs (i.e., 25%, 50%) suggest that neighborhood socioeconomic differences in the association of functional limitations and self-rated health is largely driven by those in the disadvantaged neighborhoods. This suggests that the neighborhood-level socioeconomic gradients in the link between functional limitations and self-rated health may not be linear. Rather, the magnitude of the link between functional limitations and health expectations declines drastically among individuals residing in socioeconomically disadvantaged neighborhoods, further contributing to a relatively better interpretation of their current health than those from advantaged neighborhoods. Together, our sensitivity analyses suggest that the underestimated neighborhood disparities in self-rated health is amplified with the presence of severe functional limitations, and specifically among individuals residing in the disadvantaged neighborhoods.

It is important to note several study limitations. First, this study is based on a cross-sectional study design, thus the directionality/causal relationship between the variables is unclear. Moreover, we used self-rated data to measure functional limitation and chronic conditions, which are subject to recall and respondent bias. In addition, our measure of chronic conditions did not account for the level of disease severity. Moreover, because this study is based on a cross-sectional study design, we cannot infer that neighborhood context causes functional limitations. Further studies examining the long-term neighborhood effect on healthier older adults would be needed to better understand the mechanism of neighborhood context effects on functional limitation. Furthermore, as individuals who dropped out after the first wave of MIDUS have been found to have combinations of vulnerability factors including poor health and low income [31], there is possibility of attrition and survivorship bias of the current sample as the study used later waves of data from the main MIDUS sample. Lastly, while the current study does include perceived neighborhood quality as a control variable, other qualitative measures of the neighborhood environment such as quality of social services were not available in the current dataset. Following our findings in line with the social comparison theory, lack of information on the health of peers within the neighborhood also limits directly testing this perspective. Future research on how such qualitative measures relate to socioeconomic disparities in self-rated health will benefit our understanding and use of self-rated health as a proxy for health risks.

Beyond such limitations, the present study is the first of its kind to take into account neighborhood-level SES in understanding the link between functional limitations and self-rated health. Our findings suggest that how one’s physical functioning is related to perceived health status is conditional of one’s neighborhood SES, supporting the idea that self-rated health should be explicated through the lens of one’s environmental context. The study also adds to existing research on how neighborhood differences in self-assessed health status is underestimated, particularly showing that such underestimation increases with severe functional limitations. Thus, when interpreting self-rated health status, values should not be taken face value, and should be considered along with the environmental conditions of where one resides.

## Supporting information

S1 Table Sensitivity analysis using ADL severity. (DOCX)

S2 Table Sensitivity analysis by different SDI cutoff to define neighborhood disadvantage. (DOCX)

S3 Table Sensitivity analysis using binary (low vs. high) self-rated health. (DOCX)
