# Supplementary material for: The moderating role of neighborhood disadvantage on the link between functional limitations and self-rated health
Source: PLoS One. 2023 Apr 5;18(4):e0283796. doi: 10.1371/journal.pone.0283796 (PMC10075450; doi:10.1371/journal.pone.0283796)
Supplement: S1 Table — (DOCX) [file pone.0283796.s001.docx]

**Supporting Information**

**S1 Table. Sensitivity Analysis Using ADL Severity.**

|  | B | SE B | F | p-value |
| --- | --- | --- | --- | --- |
| ADL Severity (mean-centered) | -0.69 | 0.04 | -19.06 | <0.001 |
| Neighborhood Disadvantage (Reference: Most Disadvantaged Neighborhoods) | -0.04 | 0.05 | -0.83 | 0.405 |
| Age (mean-centered) | 0.27 | 0.02 | 14.12 | <0.001 |
| Gender (Reference: Male) | 0.33 | 0.04 | 9.00 | <0.001 |
| Race/Ethnicity (Reference: White) |  |  |  |  |
| *Black* | -0.01 | 0.06 | -0.13 | 0.897 |
| *Other* | 0.03 | 0.07 | 0.38 | 0.706 |
| Education (Reference: HS/GED or lower) |  |  |  |  |
| *Some College* | -0.03 | 0.05 | -0.65 | 0.515 |
| *Bachelor's degree or higher* | 0.07 | 0.05 | 1.55 | 0.121 |
| Perceived Neighborhood Quality (mean-centered) | 0.09 | 0.02 | 4.59 | <0.001 |
| Number of Chronic Illnesses (mean-centered) | -0.36 | 0.02 | -17.47 | <0.001 |
| ADL severity x Neighborhood Disadvantage | -0.14 | 0.04 | -3.56 | <0.001 |

Note: ADL=Activities of Daily Living(s); HS/GED = high school or General Educational Development
