# Supplementary material for: The moderating role of neighborhood disadvantage on the link between functional limitations and self-rated health
Source: PLoS One. 2023 Apr 5;18(4):e0283796. doi: 10.1371/journal.pone.0283796 (PMC10075450; doi:10.1371/journal.pone.0283796)
Supplement: S2 Table — (DOCX) [file pone.0283796.s002.docx]

**S2 Table. Sensitivity Analysis by Different SDI cutoff to Define Neighborhood Disadvantage.**

|  | 50th percentile Cutoff | | | | 25th percentile Cutoff | | | |
| --- | --- | --- | --- | --- | --- | --- | --- | --- |
|  | B | SE B | F | p-value | B | SE B | F | p-value |
| Neighborhood Disadvantage (Reference: Most Disadvantaged Neighborhoods) | 0.01 | 0.08 | 0.07 | 0.944 | 0.00 | 0.08 | 0.05 | 0.958 |
| ADL Count (Reference: No ADL) |  |  |  |  |  |  |  |  |
| *1-2* | -0.46 | 0.08 | -5.67 | <0.001 | -0.47 | 0.06 | -7.21 | <0.001 |
| *3-5* | -0.87 | 0.08 | -10.28 | <0.001 | -0.85 | 0.07 | -12.55 | <0.001 |
| *6-8* | -1.34 | 0.09 | -15.39 | <0.001 | -1.33 | 0.07 | -18.31 | <0.001 |
| *9-10* | -2.13 | 0.09 | -24.73 | <0.001 | -2.14 | 0.07 | -28.90 | <0.001 |
| Age (mean-centered) | 0.28 | 0.02 | 14.08 | <0.001 | 0.28 | 0.02 | 14.08 | <0.001 |
| Gender (Reference: Male) | 0.32 | 0.04 | 8.75 | <0.001 | 0.32 | 0.04 | 8.76 | <0.001 |
| Race/Ethnicity (Reference: White) |  |  |  |  |  |  |  |  |
| *Black* | 0.03 | 0.06 | 0.50 | 0.617 | 0.03 | 0.06 | 0.47 | 0.637 |
| *Other* | 0.01 | 0.07 | 0.20 | 0.839 | 0.01 | 0.07 | 0.19 | 0.850 |
| Education (Reference: HS/GED or lower) |  |  |  |  |  |  |  |  |
| *Some College* | -0.01 | 0.05 | -0.20 | 0.844 | -0.01 | 0.05 | -0.19 | 0.848 |
| *Bachelor's degree or higher* | 0.10 | 0.05 | 2.07 | 0.04 | 0.10 | 0.05 | 2.09 | 0.04 |
| Perceived Neighborhood Quality (mean-centered) | 0.07 | 0.02 | 3.70 | <0.001 | 0.07 | 0.02 | 3.76 | <0.001 |
| Number of Chronic Illnesses (mean-centered) | -0.40 | 0.02 | -19.52 | <0.001 | -0.41 | 0.02 | -19.52 | <0.001 |
| ADL count x Neighborhood Disadvantage |  |  |  |  |  |  |  |  |
| *1-2 x* Neighborhood Disadvantage | 0.02 | 0.11 | 0.20 | 0.841 | 0.06 | 0.12 | 0.54 | 0.590 |
| *3-5 x* Neighborhood Disadvantage | 0.01 | 0.11 | 0.12 | 0.908 | -0.05 | 0.12 | -0.38 | 0.701 |
| *6-8 x* Neighborhood Disadvantage | 0.05 | 0.12 | 0.41 | 0.683 | 0.04 | 0.13 | 0.29 | 0.772 |
| *9-10 x* Neighborhood Disadvantage | -0.05 | 0.11 | -0.47 | 0.639 | -0.07 | 0.14 | -0.48 | 0.634 |

Note: ADL=Activities of Daily Living(s); HS/GED = high school or General Educational Development.
