# Supplementary material for: The moderating role of neighborhood disadvantage on the link between functional limitations and self-rated health
Source: PLoS One. 2023 Apr 5;18(4):e0283796. doi: 10.1371/journal.pone.0283796 (PMC10075450; doi:10.1371/journal.pone.0283796)
Supplement: S3 Table — (DOCX) [file pone.0283796.s003.docx]

**S3 Table. Sensitivity Analysis Using Binary (Low vs. High) Self-Rated Health.**

|  | B | SE B | z | p-value |
| --- | --- | --- | --- | --- |
| Neighborhood Disadvantage (Reference: Most Disadvantaged Neighborhoods) | 0.39 | 0.34 | 1.12 | 0.262 |
| ADL Count (Reference: No ADL) |  |  |  |  |
| *1-2* | -0.74 | 0.35 | -2.11 | 0.035 |
| *3-5* | -1.01 | 0.34 | -2.96 | 0.003 |
| *6-8* | -1.62 | 0.32 | -5.06 | <0.001 |
| *9-10* | -2.30 | 0.31 | -7.49 | <0.001 |
| Age (mean-centered) | 0.35 | 0.05 | 7.49 | <0.001 |
| Gender (Reference: Male) | 0.32 | 0.09 | 3.67 | <0.001 |
| Race/Ethnicity (Reference: White) |  |  |  |  |
| *Black* | 0.15 | 0.10 | 1.50 | 0.133 |
| *Other* | 0.49 | 0.11 | 4.50 | <0.001 |
| Education (Reference: HS/GED or lower) |  |  |  |  |
| *Some College* | -0.24 | 0.13 | -1.89 | 0.059 |
| *Bachelor's degree or higher* | -0.01 | 0.16 | -0.08 | 0.935 |
| Perceived Neighborhood Quality (mean-centered) | 0.13 | 0.04 | 3.12 | 0.002 |
| Number of Chronic Illnesses (mean-centered) | -0.45 | 0.04 | -11.20 | <0.001 |
| ADL count x Neighborhood Disadvantage |  |  |  |  |
| *1-2 x* Neighborhood Disadvantage | 0.13 | 0.43 | 0.30 | 0.764 |
| *3-5 x* Neighborhood Disadvantage | -0.50 | 0.41 | -1.22 | 0.222 |
| *6-8 x* Neighborhood Disadvantage | -0.58 | 0.38 | -1.50 | 0.133 |
| *9-10 x* Neighborhood Disadvantage | -0.85 | 0.37 | -2.31 | 0.021 |

Note: ADL=Activities of Daily Living(s); HS/GED = high school or General Educational Development.
